# Incidence and risk factors of low testosterone in infertile men with normal sperm concentration

**DOI:** 10.3389/fendo.2026.1797678

**Published:** 2026-05-01

**Authors:** Chyau-Wen Lin, Eric Yi-Hsiu Huang, William J. Huang, I-Shen Huang

**Affiliations:** 1 Department of Urology, Taipei City Hospital, Taipei, Taiwan; 2 Department of Urology, Taipei Veterans General Hospital, Taipei, Taiwan; 3 Department of Urology, National Yang Ming Chiao Tung University School of Medicine, Taipei, Taiwan; 4 Department of Urology, National Yang Ming Chiao Tung University Hospital, Yilan, Taiwan

**Keywords:** clomiphene citrate, endocrine evaluation, hypogonadism, male infertility, normozoospermia, testosterone

## Abstract

**Background:**

Current guidelines recommend endocrine evaluation for infertile men; however, those with normal sperm concentration (≥15 million/mL) may be under-referred for comprehensive assessment. This study aimed to investigate the prevalence of low testosterone in infertile men with normal sperm concentration and to determine the proportion who remain endocrinologically unevaluated.

**Methods:**

A retrospective review was conducted on infertile men with initial sperm concentration ≥15 million/mL evaluated at a single tertiary center from January 2013 to December 2020. Low testosterone was defined as serum total testosterone below 300 ng/dL. Multivariate logistic regression identified independent predictors of low testosterone.

**Results:**

Of 3,147 men meeting inclusion criteria, 77.2% (n=2,429) did not undergo hormonal evaluation. Endocrine assessment rates varied significantly by specialty: 100% for male infertility fellowship-trained urologists, 23.2% for general urologists, and 1.2% for gynecologists. Among 718 men who underwent hormonal testing, 24.1% had low testosterone. Multivariate logistic regression identified higher BMI (OR 1.083, 95% CI 1.031–1.136, p=0.001) and lower estradiol (OR 0.956, 95% CI 0.937–0.976, p<0.001) as independent predictors. Obese men had significantly higher odds of low testosterone compared with normal-weight men (OR 1.725, 95% CI 1.010–2.943, p=0.046). Of 66 treated men, significant testosterone increases were observed at all follow-up time points (all p ≤ 0.001), and six achieved natural pregnancy.

**Conclusion:**

Over three-quarters of normozoospermic infertile men did not receive hormonal evaluation. Among those evaluated, nearly one-quarter had low testosterone, with obesity identified as an independent risk factor. These findings highlight a critical screening gap and support routine endocrine screening regardless of sperm concentration, particularly in men with metabolic risk factors such as obesity, as low testosterone represents a treatable condition that may improve fertility outcomes.

## Introduction

Approximately 15% of couples encounter difficulty in achieving conception, with male reproductive issues being implicated in nearly half of these instances (1). A comprehensive andrological assessment is imperative during initial diagnosis to identify and address potential factors hindering fertility (2). Semen analysis is a pivotal component of infertile couples’ evaluation, as it provides crucial information regarding sperm concentration, motility, and morphology. However, routine semen testing does not assess the fertilization capacity of sperm or the intricate modifications that occur within a woman’s reproductive tract before conception. Furthermore, normal sperm concentration does not exclude clinically significant male factor infertility, as it does not reflect sperm functional capacity, DNA integrity, or fertilization potential; men with normozoospermia may still harbor underlying sperm functional defects including elevated DNA fragmentation, defective acrosome reaction, and abnormal chromatin packaging, all of which may contribute to infertility despite apparently normal sperm count (3). Consequently, the accuracy and precision of these parameters in determining the fertility status of men presenting to clinicians remain limited (4).

The fifth edition of the World Health Organization (WHO) laboratory manual defines oligozoospermia as sperm concentration below 15 million/mL. This threshold was derived from semen analyses of approximately 1900 fertile men across three continents whose partners achieved pregnancy within 12 months, using the fifth percentile of the one-sided lower reference distribution (5). Nevertheless, criticism regarding the use of reference ranges for semen parameters as the primary method for assessing male fertility potential led to the omission of reference ranges in the subsequent sixth edition. Instead, decision limits were employed to identify abnormal ejaculates (6). In clinical practice, the persistence of misconceptions among a considerable number of healthcare providers who continue to utilize the 15 million/mL threshold to distinguish fertile from infertile cases often results in patients believing that further evaluation is unnecessary if their sperm concentration is within the reference range.

In 2015, the American Society for Reproductive Medicine issued an updated committee opinion on the diagnosis of infertility in men (7). This committee opinion underscores the potential improvement of male partner fertility and the prospect of achieving conception via the identification and treatment of remediable conditions. Low serum total testosterone (referred to as “low testosterone”) is a significant contributor to infertility, affecting approximately 15% of infertile men (8). Moreover, low testosterone is associated with negative cardiometabolic consequences, including metabolic syndrome and increased cardiovascular disease risk (9). Consequently, the 2021 updated European Association of Urology guidelines recommend the inclusion of biochemical screening as an integral component of the diagnostic process for male infertility (2). Similarly, the American Urological Association guideline recommends that clinicians measure total testosterone in men diagnosed with infertility (10). Nonetheless, a recent study revealed that only approximately 20% of men with abnormal semen analyses, ordered by non-urologists (e.g., primary care physicians), were referred to reproductive urologists for further evaluation. Notably, men with oligospermia and azoospermia were more likely to be referred to reproductive urologists, signifying that non-urologists perceived sperm concentration as a key fertility determinant. This situation creates a significant barrier to comprehensive male reproductive healthcare (11).

We hypothesized that men with sperm concentration ≥15 million/mL would have even lower referral rates than those with lower sperm concentration, leading to underdiagnosis of low testosterone. Given that hypogonadism can be treated with hormonal therapy to improve sperm concentration, motility, and the likelihood of spontaneous conception (12, 13), this referral gap may compromise fertility potential. Accordingly, this study aimed to investigate the incidence of low testosterone in infertile men with normal sperm concentration and to determine the proportion of such men who remain endocrinologically unevaluated, particularly when initially assessed by non-urologists.

## Materials and methods

### Patients

A retrospective review was conducted on men with available semen analysis data and initial semen analysis sperm concentration ≥15 million/mL from January 2013 to December 2020 using the electronic medical record system at our institution. This study included men who were seeking counseling for infertility for the first time. Although current guidelines recommend at least two semen analyses for a comprehensive assessment of male fertility, inclusion in this study was based on the initial semen analysis result, as the primary objective was to evaluate the rate of endocrine assessment and the prevalence of low testosterone in men whose first semen analysis showed normal sperm concentration, reflecting the real-world clinical scenario in which treatment decisions and referral patterns are often guided by the initial result. Low testosterone was defined as a single serum total testosterone measurement below 300 ng/dL, consistent with the 2018 American Urological Association guideline threshold; as confirmatory repeat measurements were not uniformly available in the electronic medical records given the retrospective design, this definition represents a pragmatic clinical threshold rather than a fully confirmed endocrinological diagnosis. Men with low testosterone were further classified into primary low testosterone, defined as low testosterone with elevated LH (>8.0 mIU/mL), or secondary low testosterone, defined as low testosterone with low or inappropriately normal LH (≤8.0 mIU/mL).

**Figure 1:**
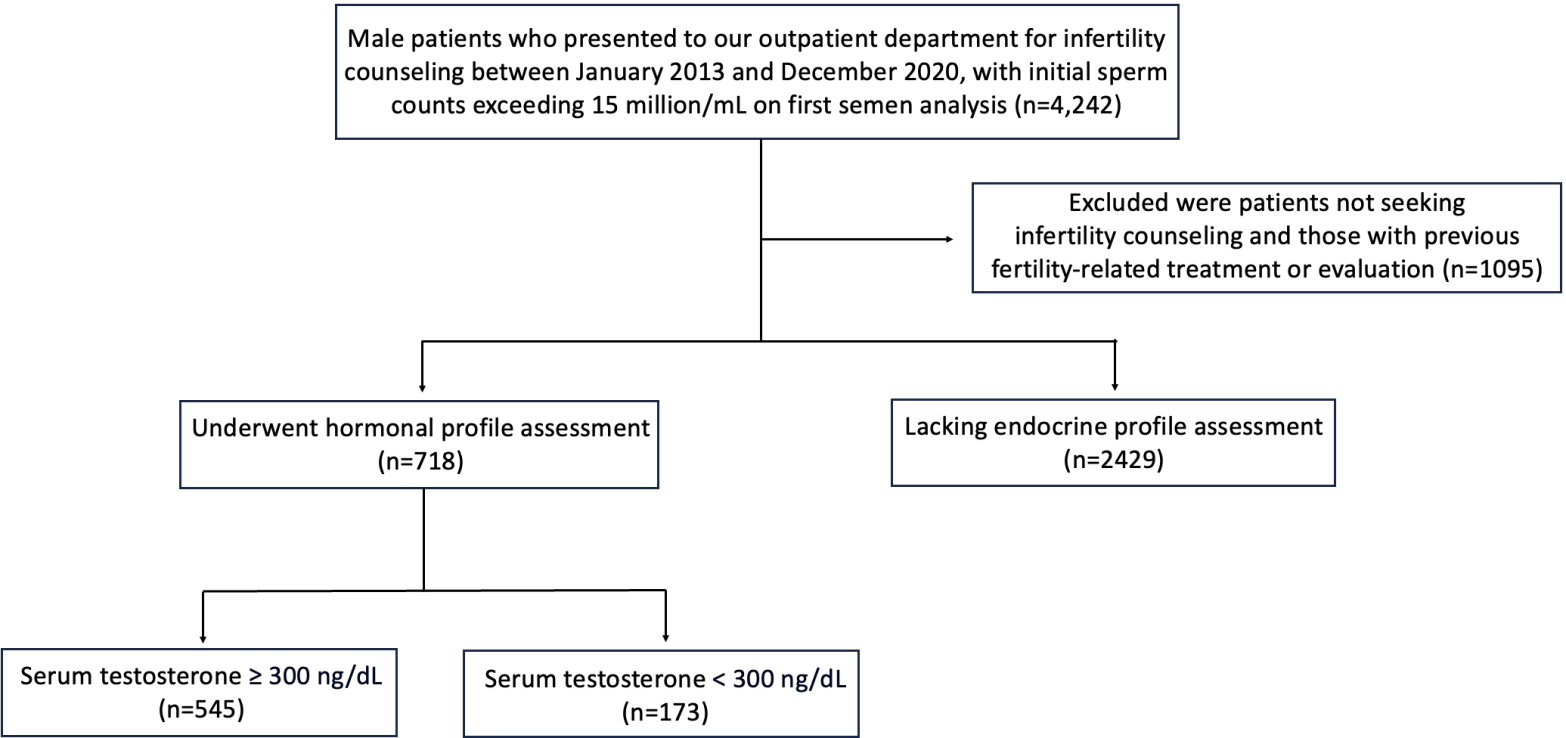
Flowchart for the selection of infertile men meeting the inclusion criteria, with an initial semen analysis showing a sperm concentration exceeding 15 million/mL.

The exclusion criteria were as follows: men (i) who did not seek counseling for infertility, (ii) underwent previous treatment or evaluation, (iii) were below 18 years of age, or (iv) had sperm concentration <15 million/mL. For further evaluation, the specialties (e.g., urology, gynecology, endocrinology, pediatrics) of physicians who prescribed semen analysis or referred these patients to urologists were documented. In instances where a thorough andrological assessment was performed by urologists who had undergone specialized male infertility fellowship training after completing their residency, a comprehensive physical examination and hormone profile assessment were routinely administered (**Figure 1**). The physical examination included testicular volume measurement using an orchidometer, varicocele assessment in the upright position and after the Valsalva maneuver according to Dubin and Amelar (14).

### Semen collection and analysis

Semen specimens were procured via masturbation after an abstinence period of 3–5 days. Subsequently, specimens were subjected to liquefaction for 30–60 min at room temperature. Parameters such as semen volume, semen pH, sperm concentration, and sperm motility were evaluated according to the WHO guidelines (15). Sperm morphology was assessed using the modified Papanicolaou staining method in accordance with the WHO guidelines for sperm examination (16).

### Blood sampling and analysis method

The serum endocrine profile, including follicle-stimulating hormone (FSH), luteinizing hormone (LH), total testosterone, prolactin, and estradiol, was analyzed using the Roche cobas^®^ 8000 automated electrochemiluminescence immunoassay (ECLIA) analyzer (Roche Diagnostics GmbH, Mannheim, Germany). All assays were performed according to the manufacturer’s instructions using Elecsys^®^ reagents. The Elecsys Testosterone II immunoassay has a measuring range of 0.087–52.0 ng/mL and a total imprecision of less than 6%, and its calibration is traceable to the isotope dilution-liquid chromatography tandem mass spectrometry (ID-LC-MS/MS) reference method per manufacturer specifications, ensuring assay standardization. Venous blood samples were collected under fasting conditions between 08:00 and 10:00 h to minimize the effect of diurnal variation on hormone levels. After collection, the blood samples were centrifuged at 1000 × g for 20 min to facilitate serum separation, and aliquots of the resultant serum were stored at −20 °C until further analysis. Institutional reference ranges for men under 50 years of age were as follows: FSH 1.3–8.4 mIU/mL, LH 1.6–8.0 mIU/mL, total testosterone 2.64–9.16 ng/mL, prolactin 4.0–15.2 ng/mL, and estradiol 11.3–43.2 pg/mL.

### Statistical analyses

Continuous variables were compared between men with normal testosterone and those with low testosterone using the Mann-Whitney U test and are presented as median (interquartile range). Categorical variables were compared using the chi-square test and are presented as number and percentage. To identify independent predictors of low testosterone, a multivariate logistic regression analysis was performed incorporating all variables achieving p<0.2 on univariate analysis. Additionally, separate logistic regression analyses were performed to evaluate the association between BMI and low testosterone: first, using BMI category as an ordinal predictor with normal-weight men (BMI 18.5–24.9 kg/m²) as the reference category, with overweight (BMI 25.0–29.9 kg/m²) and obese (BMI ≥30 kg/m²) men compared against this reference, and second, using BMI as a continuous variable to assess the odds of low testosterone per 1 kg/m² increase in BMI. The underweight group (BMI <18.5 kg/m²) was excluded from the BMI category logistic regression due to insufficient sample size (n=5). Results from all logistic regression analyses are expressed as odds ratios (OR) with 95% confidence intervals (CI). Pre- and post-treatment semen analysis results and serum hormone levels were compared using the Wilcoxon signed-rank test. All statistical analyses were performed using SPSS Statistics version 26.0 (IBM Corp., Armonk, NY, USA), with statistical significance set at a p-value of <0.05.

## Results

### Patient characteristics

**Figure 2:**
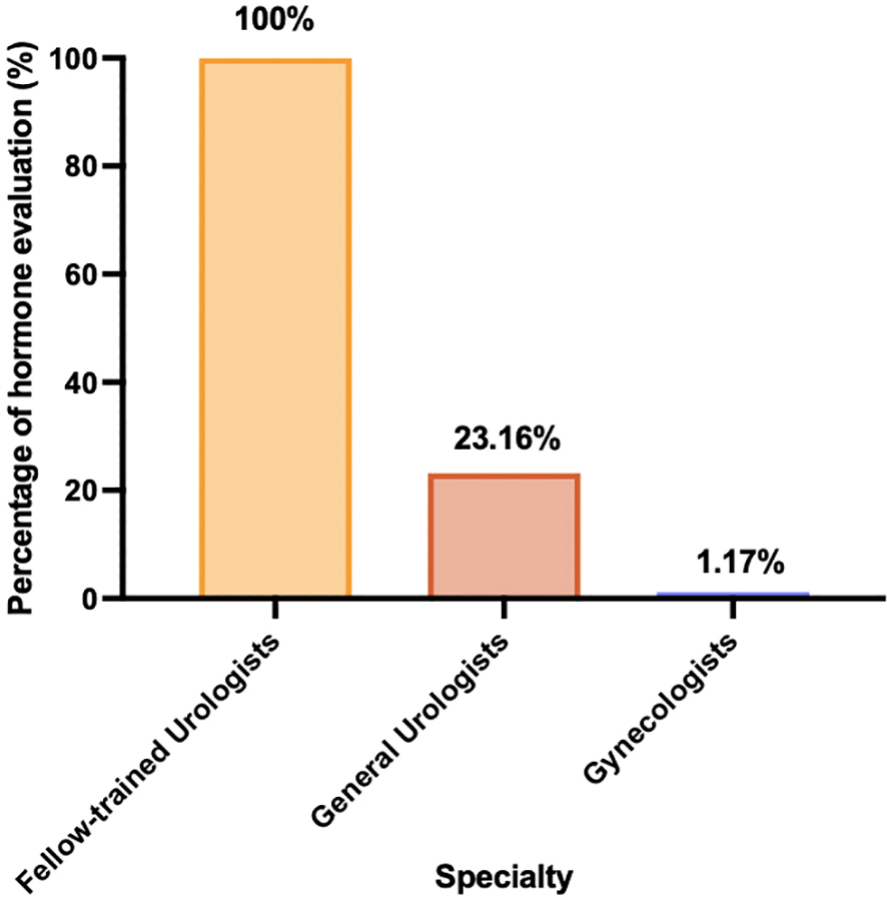
Percentage of hormone parameter evaluations by medical specialty.

A total of 5,533 patients underwent semen analysis during the study period. After applying the exclusion criteria, 3,147 patients with sperm concentration ≥15 million/mL who met the predefined inclusion criteria were retained for analysis (**Figure 1**). In this cohort, 77.2% (n=2,429) did not undergo the recommended hormone evaluation. Within this subgroup of patients without endocrine evaluation, 2,356 individuals had initially sought evaluation from gynecologists and did not receive referrals to reproductive urologists for comprehensive physical examination or endocrine status evaluation. Among 718 men with sperm concentration ≥15 million/mL who underwent endocrine assessment, evaluation was conducted by urologists in 704 patients, whereas the assessment was initiated by gynecologists in the 14 remaining patients, with these individuals being subsequently referred to reproductive urologists for further evaluation. However, 73 patients initially evaluated by general urologists without male infertility fellowship training did not undergo an endocrine workup. Additionally, 23 men who initially received management from gynecologists but did not undergo hormone evaluation were referred to reproductive urologists. Overall, the distribution of endocrine evaluations performed by male infertility fellowship-trained urologists, general urologists, and gynecologists was 100% (668/668), 23.2% (22/95), and 1.2% (28/2,384), respectively (**Figure 2**).

The clinical characteristics and hormonal parameters of men with and without low testosterone are summarized in **Table 1**. The prevalence of low testosterone, defined as serum total testosterone below 300 ng/dL, was 24.1% (173/718). No significant differences were observed in age, FSH, prolactin levels, or testis size between men with normal and low testosterone. However, LH and estradiol levels were significantly lower in the low testosterone group compared with the normal testosterone group (LH: median 3.2 mIU/mL [IQR 2.3–4.2] vs. 3.5 mIU/mL [IQR 2.5–4.9], p=0.012; estradiol: median 18.0 pg/mL [IQR 13.0–26.0] vs. 23.0 pg/mL [IQR 17.0–30.7], p<0.001, respectively). BMI was significantly higher in the low testosterone group (median 25.2 kg/m² [IQR 23.3–28.6] vs. 24.6 kg/m² [IQR 22.7–27.2], p=0.009), based on data available from 714 patients, as BMI data were missing in 3 men in the low testosterone group and 1 man in the normal testosterone group. Additionally, the prevalence of varicocele was lower in the low testosterone group (53.8% vs. 62.9%, p=0.039). Multivariate logistic regression analysis incorporating all variables with univariate p<0.2 identified BMI and estradiol as independent predictors of low testosterone. Higher BMI was independently associated with increased odds of low testosterone (OR 1.083, 95% CI 1.031–1.136, p=0.001), while higher estradiol was independently associated with decreased odds of low testosterone (OR 0.956, 95% CI 0.937–0.976, p<0.001). LH and varicocele did not reach statistical significance in the multivariate model (OR 0.955, 95% CI 0.872–1.046, p=0.320; and OR 0.729, 95% CI 0.498–1.070, p=0.106, respectively).

**Table 1:** Characteristics of infertile men with hypogonadal and eugonadal status.

Variable	Testosterone ≥ 300 ng/dL (*n* = 545)		Testosterone < 300 ng/dL (*n* = 173)		Univariate p value	OR	95% CI	Multivariate p value
	Mean (SD)	Median (IQR)	Mean (SD)	Median (IQR)				
Age, years	37.0 (5.3)	36.0 (34.0–40.0)	36.7 (6.6)	36.0 (33.0–40.0)	0.557			
BMI, kg/m^2^	25.2 (3.6)	24.6 (22.7–27.2)	26.2 (4.3)	25.2 (23.3–28.6)	0.009^**^	1.083	1.031-1.136	0.001^**^
Varicocele	343 (62.9%)		93 (53.8%)		0.039^*^	0.729	0.498-1.070	0.106
Testis size, mL	18.6 (3.5)	18.0 (16.5–20.0)	18.2 (3.1)	18.0 (17.0–20.0)	0.285			
Semen parameter								
Volume, mL	4.5 (2.0)	4.5 (3.0–5.0)	4.4 (2.1)	4.0 (3.0–5.5)	0.492			
pH	8.0 (0.3)	8.0 (8.0–8.0)	8.0 (0.3)	8.0 (8.0–8.2)	0.259			
Concentration, million/mL	90.5 (66.8)	73.0 (41.0–122.0)	98.2 (72.0)	81.0 (46.0–122.0)	0.237			
Total motility, %	53.8 (18.2)	55.0 (42.0–66.0)	53.2 (18.8)	55.0 (40.0–67.0)	0.831			
Morphology, %	5.0 (3.2)	4.2 (2.9–6.1)	5.0 (2.9)	4.3 (3.0–6.1)	0.604			
Serum hormone profile								
FSH, mIU/mL	4.9 (2.6)	4.4 (3.2–5.9)	4.8 (2.7)	4.3 (3.1–5.7)	0.501			
LH, mIU/mL	4.2 (2.7)	3.5 (2.5–4.9)	3.6 (2.0)	3.2 (2.3–4.2)	0.012^*^	0.955	0.872-1.046	0.320
Testosterone, ng/dL	513.3 (198.0)	460.0 (366.0–598.0)	234.0 (49.5)	245.0 (204.0–273.0)	<0.001^***^			
Prolactin, ng/mL	11.6 (7.2)	9.9 (7.8–13.3)	26.1 (153.8)	10.7 (7.9–13.5)	0.296			
Estradiol, pg/mL	25.8 (13.5)	23.0 (17.0–30.7)	20.6 (10.0)	18.0 (13.0–26.0)	<0.001^***^	0.956	0.937-0.976	<0.001^***^

Values are reported as means (SD, standard deviations), medians (IQR, interquartile range) or numbers (percentages).

BMI, body mass index; FSH, follicle-stimulating hormone; LH, luteinizing hormone; OR, odds ratio; CI, confidence interval.

BMI data were available in 714 of 718 patients (3 missing in the low testosterone group and 1 in the normal testosterone group).

* p<0.05; ** p<0.01; *** p<0.001; **** p<0.0001.

Bold type indicates statistically significant *p*-values.

Further stratification by BMI category among the 714 men with available BMI data revealed a progressive increase in the prevalence of low testosterone: 20.0% (1/5) in underweight men, 21.2% (80/378) in normal-weight men, 25.4% (64/252) in overweight men, and 31.6% (25/79) in obese men, although the overall chi-square test across all four categories did not reach statistical significance (p=0.210) (**Table 2**). Logistic regression analysis using normal-weight men as the reference category and excluding the underweight group due to insufficient sample size (n=5) demonstrated that overweight men showed a non-significant trend toward higher odds of low testosterone (OR 1.268, 95% CI 0.871–1.847, p=0.216), while obese men had significantly increased odds of low testosterone compared with normal-weight men (OR 1.725, 95% CI 1.010–2.943, p=0.046). When BMI was analyzed as a continuous variable, each 1 kg/m² increase in BMI was independently associated with a 6.9% increase in the odds of low testosterone (OR 1.069, 95% CI 1.023–1.118, p=0.003) (**Table 3**).

**Table 2:** Prevalence of low testosterone by BMI category among normozoospermic infertile men (n=714).

BMI category	Total	Testosterone ≥ 300 ng/dL	Testosterone < 300 ng/dL	Prevalence
Underweight (< 18.5 kg/m^2^)	5	4	1	20.0%
Normal (18.5–24.9 kg/m^2^)	378	298	80	21.2%
Overweight (25–29.9 kg/m^2^)	252	188	64	25.4%
Obese (≥30 kg/m^2^)	79	54	25	31.6%

BMI, body mass index.

**Table 3:** Logistic regression analysis of BMI categories and continuous BMI as predictors of low testosterone in normozoospermic infertile men.

Category	OR	95% CI	p value
Normal weight (18.5–24.9 kg/m^2^)	Ref	–	–
Overweight (25–29.9 kg/m^2^)	1.268	0.871-1.847	0.216
Obese (≥30 kg/m^2^)	1.725	1.010-2.943	0.046^*^
BMI continuous (per 1 kg/m²)	1.069	1.023-1.118	0.003^**^

Analysis was performed on 709 patients with available BMI data after exclusion of the underweight group and 4 patients with missing BMI data. The continuous BMI row represents the odds of low testosterone per 1 kg/m² increase in BMI, modeled as a separate logistic regression analysis.

OR, odds ratio; CI, confidence interval; Ref, reference category; BMI, body mass index. Bold type indicates statistically significant p-values. * p<0.05; ** p<0.01.

Among the 173 men with low testosterone, LH data were available in 166 patients, with 7 men having missing LH values. Based on our institutional LH upper normal limit of 8.0 mIU/mL, primary low testosterone, defined as low testosterone with elevated LH above the upper normal limit (LH >8.0 mIU/mL), was identified in 6 men (3.6%), while secondary low testosterone, defined as low testosterone with low or inappropriately normal LH (LH ≤8.0 mIU/mL), was identified in 160 men (96.4%). The six men with primary low testosterone had LH values ranging from 8.16 to 13.91 mIU/mL, with a median LH of 9.27 mIU/mL (IQR 8.55–12.40), while men with secondary low testosterone had a median LH of 3.17 mIU/mL (IQR 2.23–4.14).

### Effects of clomiphene citrate on the endocrine profile and semen parameters

Out of 168 patients with low testosterone, 66 were treated with clomiphene citrate (50 mg) every other day. This course of action was decided upon after shared decision-making. One participant was treated with both clomiphene citrate and anastrozole because of an elevated E2/testosterone ratio. Specialists in reproductive urology were primarily responsible for prescribing these treatments to 64 patients, whereas general urologists issued the two remaining prescriptions. After undergoing the proposed treatment, an increase in plasma testosterone levels was apparent in nearly all participants. The median post-treatment testosterone levels were 637 (interquartile range [IQR]: 474 – 790) ng/dL, 647 (IQR: 478 – 879) ng/dL, 733 (IQR: 539 – 897) ng/dL, and 609 (IQR: 400–777) ng/dL at 1-, 3-, 6-, and 12-month follow-up, respectively. Additionally, the increase in testosterone levels was found to be significant, with median increments of 430 ng/dL (IQR: 202 – 572, *p* < 0.001), 468 ng/dL (IQR: 219 – 583, *p* = 0.001), 486 ng/dL (IQR: 282 – 663, *p* = 0.001), and 340 ng/dL (IQR: 218 – 416, *p* = 0.001) at corresponding intervals of 1, 3, 6, and 12 months. The patients started the study with a median baseline sperm concentration of 65.5 million/mL (IQR: 34.3 – 99.3). After treatment, their sperm concentration did not show notable changes at 1 and 3 months, maintaining levels comparable to baseline values. However, in a subset of 23 patients who sustained treatment until the 6-month mark, an increase in sperm concentration was documented in 12 patients, with a consequential median post-treatment level reaching 85 million/mL. The median increase was 21 million/mL (IQR: 0 – 52.5, *p* = 0.011). Although the observed increase in sperm concentration was statistically significant, variations were observed within the sample, with a small group of four patients displaying a decrease in sperm concentration. Analysis of parameters such as sperm motility and morphology revealed that the medical treatment initiated did not cause any significant alterations. Among 66 men with low testosterone who received clomiphene as a hormone-modulating agent, six patients achieved natural pregnancy within a median of 4 months after treatment (range: 1–14 months), resulting in four live births (**Table 4**).

**Table 4:** Changes in semen parameters and endocrine profile after hormone manipulation.

Variable	Time from initiation of clomiphene citrate treatment					*P* value (compared with baseline data)			
	Baseline (*n* = 44)	1 month after treatment (*n* = 35)	3 months after treatment (*n* = 21)	6 months after treatment (*n* = 23)	12 months after treatment (*n* = 10)	1 month after treatment	3 months after treatment	6 months after treatment	12 months after treatment
Sperm concentration, million/mL	65.5 (34.3-99.3)	76 (23.5-103)	50 (28-93)	85 (59.5-120)	130 (32.9-232)	0.903	0.390	**0.011**	0.508
Motility, %	43 (32.8-58)	47 (32-58)	42 (33-57.5)	50 (35-66.5)	41 (30.5-48.8)	0.118	0.341	0.088	0.074
Normal form, %	5.5 (3.4-6.3)	5.3 (3.1-4.9)	5.3 (3-6.1)	5.3 (3.3-7)	5.3 (2.3-6.2)	0.437	0.383	0.850	0.413

Variable	Time from initiation of clomiphene citrate treatment					*P* value (compared with baseline data)			
	Baseline (*n* = 44)	1 month after treatment (*n* = 41)	3 months after treatment (*n* = 17)	6 months after treatment (*n* = 12)	12 months after treatment (*n* = 11)	1 month after treatment	3 months after treatment	6 months after treatment	12 months after treatment
Testosterone, ng/dL	220 (192-257)	637 (474-790)	647 (478-879)	733 (539-897)	609 (400-666)	**<0.001**	**0.001**	**0.001**	**0.003**
LH, mIU/mL	3.4 (2.6-4.6)	7.8 (5-10.8)	5.2 (4.7-12.0)	6.9 (5.1-9.6)	5.2 (5.1-8.7)	**<0.001**	**0.037**	**0.008**	0.069
Estradiol, pg/mL	18 (14-27)	44.4 (35.4-61.5)	53.9 (24.4-71.8)	33.6 (24.8-61.3)	31.3 (19-58.5)	**<0.001**	**0.002**	**0.013**	0.499

Values are reported as median (IQR, interquartile range). p-values represent comparisons between each post-treatment time point and baseline using the Wilcoxon signed-rank test.

LH, luteinizing hormone; IQR, interquartile range. Bold type indicates statistically significant *p*-values (p<0.05).

SD, standard deviation; LH, luteinizing hormone.

Bold type indicates statistically significant *p*-values.

## Discussion

Our study revealed that a significant proportion of men with fertility concerns, characterized by sperm concentration >15 million/mL, initially sought consultation with gynecologists. However, a substantial majority of these men, who were part of infertile couples, did not undergo endocrine evaluation by gynecologists. Additionally, only a small proportion of these men were referred to reproductive urologists for further assessment. Intriguingly, our findings also indicated that up to 23.4% of men who underwent hormone checkups exhibited low testosterone, underscoring the importance of comprehensive and clinically integrated care for infertile couples, which can be effectively achieved through joint male- and female-factor reproductive health consultations.

Our study highlights that men initially seen by gynecologists or general urologists often do not receive male-factor infertility investigations, including endocrine evaluations. These findings suggest that some healthcare providers may prioritize sperm concentration as a key criterion for distinguishing fertile from infertile individuals, consistent with prior reports of low referral rates for normozoospermic men (11). As a result, the under-referral of these men to reproductive urologists for comprehensive assessments limits the identification and treatment of remediable conditions, such as low testosterone, posing a significant barrier to the provision of comprehensive care.

The American Society for Reproductive Medicine (7), the European Association of Urology (2), and Barratt et al. (17) all recommend that the initial screening evaluation of a male partner in an infertile couple should encompass a reproductive history assessment and analysis of at least one semen sample. If the initial evaluation yields abnormal results, referral to a specialist experienced in male reproductive health is advised. Male-factor infertility evaluation serves four primary purposes—namely, (i) to identify reversible causes of male infertility, (ii) to uncover genetic abnormalities that could potentially lead to unfavorable reproductive outcomes or genetic conditions in offspring, (iii) to elucidate reproductive options for couple, and (iv) to aid in detecting health conditions that could affect a man’s overall well-being.

The existing literature highlights several reversible causes of male infertility, including varicocele, endocrine dysfunction, and genital tract infections, among men undergoing infertility evaluation. Clomiphene citrate (CC) offers a potential intervention for addressing these conditions, particularly low testosterone. By occupying estrogen receptors in the hypothalamus and pituitary gland, it stimulates the hypothalamic–pituitary–gonadal axis, promoting the release of gonadotropins and improving testosterone production. Although some studies have suggested potential benefits in improving semen parameters, the evidence remains controversial (18–21). The most comprehensive randomized clinical trial by the WHO demonstrated no significant improvement in pregnancy rates or sperm concentrations when comparing clomiphene citrate to a placebo at 6-month follow-up (22). A recent meta-analysis of 15 studies using clomiphene citrate for male infertility showed higher sperm concentration and total sperm motility but no improvement in sperm morphology (23). However, these studies have primarily focused on oligospermic or normogonadotropic infertile men, with limited research on hormone manipulation in normospermic men with low testosterone. In contrast to prior studies reporting sperm concentration improvements after approximately 3 months of therapy, our study observed significant improvements in sperm concentration at 6 months post-treatment in normospermic men with low testosterone, suggesting that longer treatment durations may be required to optimize fertility outcomes.

The observed difference in varicocele prevalence between men with testosterone < 300 or ≥ 300 ng/dL likely reflects the distinct pathophysiological mechanisms underlying male infertility. In eugonadal men, varicocele is a primary contributor to fertility challenges, potentially inducing testicular damage through mechanisms such as increased venous reflux, localized temperature elevation, and oxidative stress. In contrast, hypogonadal men experience fertility issues predominantly driven by testosterone deficiency, with varicocele playing a less significant role. This distinction underscores the complex and heterogeneous nature of male infertility, revealing that the underlying causes can vary substantially between different patient subgroups, with anatomical vascular issues being more prominent in eugonadal men, and hormonal imbalances playing a more critical role in hypogonadal men with normospermic infertility.

Among the 718 men who underwent hormonal evaluation, the prevalence of low testosterone, defined as a single serum total testosterone measurement below 300 ng/dL, was 24.1% (173/718). It should be noted that this figure is restricted to the tested subgroup and cannot be directly extrapolated to the broader cohort of 3,147 normozoospermic infertile men, given that hormonal evaluation was not performed uniformly across all patients. Furthermore, this prevalence should be interpreted as a pragmatic clinical estimate rather than a fully confirmed endocrinological diagnosis.

The prevalence of low testosterone observed in our cohort is consistent with prior reports of androgen deficiency in infertile men. Bobjer et al. (9). reported biochemical hypogonadism in 34% of young subfertile men with sperm concentration below 20 million/mL, with the highest prevalence of 53% observed in those with nonobstructive azoospermia. While their study focused on men with abnormal semen parameters, our finding of low testosterone in 24.1% of normozoospermic infertile men demonstrates that androgen deficiency is not confined to those with overtly impaired spermatogenesis and may be substantially underrecognized in men whose sperm concentration falls within the reference range. Notably, Bobjer et al. demonstrated that hypogonadism in subfertile men was associated with measurable metabolic consequences including elevated HbA1c and reduced bone mineral density, lending additional clinical urgency to our observation that 77.2% of normozoospermic infertile men did not undergo any endocrine evaluation.

The predominance of secondary low testosterone in our cohort (96.4%), characterized by low or inappropriately normal LH and preserved testicular volume, is consistent with functional rather than organic hypogonadism, where reversible suppression of the hypothalamic-pituitary-gonadal axis occurs in the absence of primary testicular pathology. The strong association between increasing BMI and low testosterone risk observed in our cohort, confirmed by both categorical logistic regression (obese vs. normal-weight: OR 1.725, p=0.046) and continuous BMI analysis (OR 1.069 per 1 kg/m², p=0.003), supports adiposity-driven aromatization of androgens to estrogens in peripheral adipose tissue as a key underlying mechanism, leading to elevated estradiol, suppressed gonadotropin-releasing hormone pulsatility, reduced LH secretion, and consequently impaired testicular testosterone production. The identification of estradiol as an independent negative predictor of low testosterone in our multivariate model (OR 0.956, p<0.001) is consistent with this mechanism.

Several constraints must be considered when interpreting our findings. First, our data were collected from a single tertiary referral center, which may not accurately represent practice patterns applicable to broader or diverse settings, such as private reproductive endocrinologist practices, and a multicenter study would be instrumental in ensuring wider applicability. Second, the retrospective nature of our study prevented us from conclusively determining why hormonal evaluations were omitted in individual cases. The decision not to perform endocrine testing may have reflected provider-level factors, such as unfamiliarity with current guidelines, an implicit reliance on sperm concentration as a surrogate for overall reproductive health, or a tendency to prioritize female-factor evaluation. Equally, patient-level factors, including refusal, financial constraints, or limited follow-up compliance, may have contributed to the under-evaluation observed in this cohort. Although the observed specialty-based disparities in evaluation rates are suggestive of a provider-level influence, these explanations remain associative rather than definitive. Third, the clomiphene citrate cohort was small (n=66 of 173 hypogonadal men), non-randomized, and lacked a concurrent control group, precluding definitive conclusions regarding treatment efficacy. The decision to initiate clomiphene citrate was based on shared decision-making between the treating physician and each patient, introducing potential selection bias. Furthermore, follow-up semen parameter data were available in only a subset of treated patients, with 6-month data limited to 23 individuals, reflecting the high attrition inherent to retrospective real-world data. Accordingly, the clomiphene citrate findings should be regarded as hypothesis-generating rather than conclusive. Fourth, several potentially relevant covariates were not systematically captured, including lifestyle factors such as smoking, alcohol consumption, metabolic comorbidities, medication use, and psychological stress, as well as genetic screening data including karyotype and Y chromosome microdeletion status. As current guidelines reserve genetic evaluation primarily for men with severe oligozoospermia or azoospermia, these assessments were not routinely performed in our normozoospermic cohort, and residual confounding from these unmeasured variables cannot be excluded. Fifth, only total testosterone was measured in this study, and low testosterone was defined based on a single measurement without confirmatory repeat testing as recommended by the Endocrine Society, EAU, and AUA guidelines. While free testosterone and SHBG measurements were not systematically performed, current AUA guidelines reserve these assessments for cases where total testosterone is borderline or SHBG abnormality is clinically suspected. Men with borderline total testosterone but genuinely low free testosterone may have been misclassified as eugonadal in our analysis, suggesting that our reported prevalence of 24.1% is more likely a conservative estimate. Sixth, given that only 718 of 3,147 normozoospermic infertile men (22.8%) underwent hormonal evaluation, the observed prevalence of low testosterone may reflect a degree of selection bias. Men who underwent endocrine assessment were not randomly selected from the broader cohort, and the tested subgroup may not be fully representative of the overall normozoospermic infertile population. This should be borne in mind when interpreting the reported prevalence, which may not be directly generalizable to the broader population of normozoospermic infertile men. Finally, given the exploratory nature of the univariate comparisons, no formal correction for multiple testing was applied, and univariate findings should be interpreted as hypothesis-generating rather than confirmatory, with the multivariate logistic regression representing the primary inferential analysis. Collectively, future prospective multicenter studies with standardized hormonal assessment protocols, confirmatory repeat testosterone measurements, free testosterone calculation, structured assessment of provider and patient-level barriers to endocrine evaluation, and adequately powered randomized controlled trials comparing clomiphene citrate, metabolic interventions such as weight loss and exercise, and combination approaches in overweight and obese normozoospermic infertile men with low testosterone are warranted to more rigorously characterize the burden and optimal management of low testosterone in this population.

Our real-world study identified a critical gap in male reproductive healthcare: over 70% of normozoospermic men in infertile couples did not undergo hormonal evaluation. The majority of initial consultations were conducted by gynecologists, which may have contributed to the underassessment of male endocrine factors. Among those who received hormonal testing, approximately one-quarter were diagnosed with low testosterone. Treatment with clomiphene citrate demonstrated preliminary evidence of hormonal benefit, with significant increases in serum testosterone observed from the first month and sustained up to 12 months, and improvement in sperm concentration noted in a subset of patients who continued treatment beyond 6 months. However, given the small sample size and retrospective nature of the treatment analysis, these findings should be regarded as hypothesis-generating rather than conclusive. These findings underscore the importance of comprehensive endocrine evaluation in male infertility management and the potential of targeted hormonal interventions such as clomiphene citrate to improve reproductive outcomes in normozoospermic men with low testosterone. In particular, normozoospermic infertile men with elevated BMI should be considered a high-priority group for endocrine evaluation, as our data demonstrate a progressive increase in low testosterone prevalence with increasing adiposity and independently higher odds of low testosterone in obese men compared with their normal-weight counterparts.

## Data Availability

The raw data supporting the conclusions of this article will be made available by the authors, without undue reservation.
